# The predictors of necrotizing enterocolitis in newborns with low birth weight: A retrospective analysis

**DOI:** 10.1097/MD.0000000000028789

**Published:** 2022-02-18

**Authors:** Xuerong Tan, Yunxia Zhou, Lan Xu, Li Zhang, Jiaying Wang, Wenqiong Yang

**Affiliations:** a Department of Neonatology, Ya’an People's Hospital, Ya’an, Sichuan, PR China; b Nursing Department, Ya’an People's Hospital, Ya’an, Sichuan, PR China.

**Keywords:** infants, low birth weight, necrotizing enterocolitis, prevention, treatment

## Abstract

There are needs to investigate the influencing factors of necrotizing enterocolitis (NEC) in low birth weight (LBW) newborns, to provide insights into the clinical management of NEC.

This study was a retrospective cohort study. Infants admitted to our hospital from January 1, 2019 to June 30, 2021 were selected. The clinical characteristics of NEC and no-NEC infants were evaluated. Logistic regression analyses were conducted to assess the risk factors of NEC in LBW infants.

A total of 192 LBW infants were included, the incidence of NEC in LBW infants was 35.42%. There were significant differences in the congenital heart disease, sepsis, breastfeeding, blood transfusion and probiotics feeding between NEC and no-NEC group (all *P* < .05), and there were no significant differences in birth weight, gestational age, mother's pregnancy-induced hypertension, premature rupture of fetal membrane, amniotic fluid pollution, fetal asphyxia, neonatal respiratory distress syndrome and mechanical ventilation between NEC and no-NEC group (all *P* > .05). Congenital heart disease (OR: 2.128, 95% CI: 1.103–3.511), sepsis (OR: 1.630, 95% CI: 1.022–2.549), and blood transfusion (OR: 1.451, 95% CI: 1.014–2.085) were the independent risk factors for NEC in LBW infants, and breastfeeding (OR: 0.494, 95% CI: 0.023–0.928), probiotics feeding (OR: 0.816, 95% CI: 0.782–0.982) were the protective factors for the NEC in LBW infants. The prognosis of NEC infants undergone surgery treatment was better than that of infants undergone conservative treatments (*P* = .043).

The incidence of NEC in LBW is high, which is affected by many factors, and comprehensive interventions targeted on the risk and protective factors should be made to improve the prognosis of LBW infants.

## 1 Introduction

Birth weight is an important indicator that reflects the fetal nutrition and growth and development in the mother's womb.^[1]^ It is also an important indicator to measure the social development of a country and region and everyone's access to primary health care.^[2]^ Low birth weight has become the leading cause of death for children under 5 years of age in China, seriously threatening children's life, health and intellectual development.^[3]^ Improving the prognosis of low birth weight (LBW) infants and reducing the mortality of low birth weight infants are urgent and critical to the healthy career worldwide. With the advancement of medical technology, the survival rate of infants with low birth weight has much improved.^[4]^ However, due to their weak metabolic and immune functions, they are susceptive to various complications. The incidence of necrotizing enterocolitis (NEC) in preterm infants is 2% to 7.5% as per national prospective surveillance study^[5]^ and 5% to 22% in extremely low birth weight babies.^[6]^ The prevention and treatment of complications associated with the low birth weight is vital to the prognosis of infants.

NEC is one of the common diseases that seriously threaten the life of newborns. The morbidity and mortality are higher in premature infants with LBW or gestational age less than 36 weeks.^[7]^ In recent years, the rapid development of perinatal science and neonatal intensive care treatment technology have significantly improved the survival of low birth weight or premature infants.^[8]^ Although the symptoms of NEC can be relieved through conservative treatments such as fasting, parenteral nutrition, fluid resuscitation, antibiotics, and maintaining acid-base balance, there are still a considerable number of children with NEC that require surgical treatment.^[^9^,^^10^^]^ Therefore, it is necessary to further explore the clinical features and influencing factors of NEC in low birth weight infants, and provide evidence support for the prevention and treatment of clinical NEC.

## 2 Methods

In this study, all methods were performed in accordance with the relevant guidelines and regulations. This study was a retrospective cohort study. The study was approved by the institutional review boards of our hospital (approval number:1800102), and written informed consents had been obtained from the relatives of included infants.

### 2.1 Study population

This study selected LBW infants admitted to our hospital from January 1, 2019 to June 30, 2021 as the research population. The inclusion criteria of this study were: the birth weight of the newborn was 1500 to 2500 g; the case data were complete, and the relevant guardians had signed the informed consent. The exclusion criteria for this study were: incomplete data; infants who was diagnosed with congenital intestinal atresia, megacolon, malrotation, anal atresia; infants whose guardians did not agree to participate in this study.

### 2.2 The diagnosis of NEC

The diagnosis of NEC was made in comply with the relevant diagnostic criteria.^[^11^,^^12^^]^ The infants should have 2 or more of the following 4 characteristics and be diagnosed by X-ray examination:

1. abdominal distension;
2. blood in the stool;
3. lethargy, apnea, low muscle tension;
4. Gas in the intestinal wall.

And the diagnosis of NEC was made by 2 of experienced doctors in our departments.

### 2.3 Data collection

The 2 authors used self-designed forms to collect relevant clinical data of the children, including: birth weight, Gestational age, gender, whether the mother had pregnancy-induced hypertension, premature rupture of fetal membrane, amniotic fluid pollution, fetal asphyxia, congenital heart disease, neonatal respiratory distress syndrome, mechanical ventilation, sepsis, breastfeeding, blood transfusion, probiotics feeding.

### 2.4 Treatment

Infants with following conditions were treated with surgery, including pneumoperitoneum, positive abdominal puncture, or progressively worsening NEC symptoms and signs (such as signs of peritonitis, persistent blood in the stool, abdominal X-ray showing large amounts of ascites, fixed intestinal loop or continuous decrease in platelets, or continuous increase in white blood cells, etc.) the surgical methods were one-stage enterectomy, enterostomy, enterostomy, or abdominal drainage. Other NEC infants received conservative medical treatment.

### 2.5 Statistical methods

SPSS 23.0 statistical software was used for data analysis, measurement data was expressed as x ± s, and *t* test was used for data comparison between the 2 groups. The enumeration data was expressed as a percentage (%). For the analysis of clinical influencing factors in children with NEC, the NEC group and the no-NEC group were firstly subjected to univariate analysis, and then factors with statistically significant differences in the univariate analysis were included in the multivariate logistic regression analysis. Chi-Squared test or Fisher exact test were performed to evaluate the group difference. Please clarify. The test level α was 0.05, and the difference was statistically significant if *P* < .05.

## 3 Results

### 3.1 LBW infants

A total of 192 LBW infants were included in this present study, of whom 68 infants had been diagnosed as NEC, the incidence of NEC in LBW infants was 35.42%. As presented in Table 1, there were significant differences in the congenital heart disease, sepsis, breastfeeding, blood transfusion and probiotics feeding between NEC and no-NEC group (all *P* < .05), and there were no significant differences in birth weight, gestational age, mother's pregnancy-induced hypertension, premature rupture of fetal membrane, amniotic fluid pollution, fetal asphyxia, neonatal respiratory distress syndrome and mechanical ventilation between NEC and no-NEC group (all *P* > .05).

**Table 1:** The characteristics of included LBW infants.

Variables	NEC group (n = 68)	No-NEC group (n = 124)	t/χ^2^	*P*
Birth weight (g)	1838.12 ± 16.45	1840.22 ± 13.39	12.761	.067
Gestational age (weeks)	30.54 ± 1.19	31.02 ± 2.24	2.197	.061
Male/female	36/32	66/58	1.291	.085
Mother's pregnancy-induced hypertension	12 (17.65%)	20 (16.13%)	1.744	.092
Premature rupture of fetal membrane	19 (27.94%)	32 (25.81%)	1.602	.077
Amniotic fluid pollution	30 (44.12%)	55 (44.35%)	1.812	.075
Fetal asphyxia	10 (14.71%)	15 (12.10%)	1.097	.056
Congenital heart disease	11 (16.18%)	9 (7.26%)	1.182	.039
NRDS	10 (14.71%)	16 (12.90%)	1.201	.066
Mechanical ventilation	18 (26.47%)	28 (22.58%)	1.102	.053
Sepsis	16 (23.53%)	10 (8.06%)	1.614	.008
Breastfeeding	32 (47.06%)	105 (84.67%)	1.126	.012
Blood transfusion	12 (17.65%)	7 (5.65%)	1.229	.005
Probiotics feeding	11 (16.18%)	60 (48.38%)	1.176	.014

### 3.2 Risk factors of NEC in LBW infants

The variable assignments of multivariate logistic regression were showed in Table 2. As presented in Table 3, Logistic regression analyses indicated that congenital heart disease (OR: 2.128, 95% CI: 1.103–3.511), sepsis (OR: 1.630, 95% CI: 1.022–2.549), and blood transfusion (OR: 1.451, 95% CI: 1.014–2.085) were the independent risk factors for NEC in LBW infants, and breastfeeding (OR: 0.494, 95% CI: 0.023–0.928), probiotics feeding (OR: 0.816, 95% CI: 0.782–0.982) were the protective factors for the NEC in LBW infants.

**Table 2:** The variable assignment of multivariate logistic regression.

Factors	Variables	Assignment
NEC	Y	Yes = 1, No = 2
Congenital heart disease	X_1_	Yes = 1, No = 2
Sepsis	X_2_	Yes = 1, No = 2
Breastfeeding	X_3_	Yes = 1, No = 2
Blood transfusion	X_4_	Yes = 1, No = 2
Probiotics feeding	X_5_	Yes = 1, No = 2

**Table 3:** Logistic regression analysis on the risk factors of NEC in LBW infants.

Variables	β	Wald	OR	95% CI	*P*
Congenital heart disease	0.118	6.163	2.128	1.103–3.511	.041
Sepsis	0.122	2.199	1.630	1.022–2.549	.026
Breastfeeding	0.108	2.507	0.494	0.023–0.928	.021
Blood transfusion	0.167	3.949	1.451	1.014–2.085	.014
Probiotics feeding	0.193	1.099	0.816	0.782–0.982	.016

### 3.3 The prognosis of NEC treatment

Of the 68 LBW infants, 32 infants underwent surgical treatment. 20 underwent enterostomy, 9 infants underwent one-stage enterectomy, and 3 infants underwent abdominal drainage. Thirty-six patients underwent conservative treatments. As indicated in Table 4, the prognosis of NEC infants undergone surgery treatment was better than that of infants undergone conservative treatments (*P* = .043).

**Table 4:** The prognosis of NEC treatments of LBW infants (n = 68).

			Death		
Treatment	Cases	Cure	Multiple organ failure	Severe sepsis	Give up
Surgery	32	29 (90.63%)	2 (6.25%)	1 (3.12%)	0 (0%)
Conservative treatments	36	28 (77.78%)	4 (11.11%)	0 (0%)	4 (11.11%)
χ^2^	1.127				
*P*	.043				

## 4 Discussions

NEC is a common intestinal severe disease in the pediatric clinical setting. It mostly occurs in sick newborns or premature infants, and has a high mortality.^[13]^ Studies^[^14^,^^15^^]^ have reported that the mortality of NEC is about 25% to 30%. The incidence of neurodevelopmental abnormalities, gastrointestinal complications, and intestinal stenosis in survivors will also increase significantly.^[^16^,^^17^^]^ Clinically, NEC-related influencing factors include intestinal infections, premature birth, local injury, and ischemia and hypoxia, which may destroy the intestinal mucosa and hinder its blood supply, leading to intestinal mucosal ischemia and hypoxia damage.^[18]^ The occurrence of NEC in LBW infants is difficult to distinguish by clinical manifestations. Because of its poor specificity, blood in the stool, abdominal distension, and vomiting are rarely seen in the early stage.^[19]^ Once it occurs, it is most likely to be late and it often accompanied by intestinal perforation.^[20]^ Therefore, the treatment of NEC in children with low birth weight is more difficult and the prognosis is poor. Even through our study is a single center study experience, the results of our study have found that the incidence of NEC in LBW infants was 35.42%, and congenital heart disease, sepsis, and blood transfusion were the independent risk factors for NEC in LBW infants, and breastfeeding, probiotics feeding were the protective factors for the NEC in LBW infants, early preventions targeted on the risk factors are warranted.

Congenital heart disease can affect the blood flow of the intestinal tract. Due to the poor blood circulation, shock is prone to occur.^[21]^ The abdominal aortic blood reflux occurs during diastole, resulting in insufficient intestinal blood flow and the mesentery in a hypoxic-ischemic state.^[22]^ The high levels of TNF-α and IL-3 in children with congenital heart disease are also a factor that causes intestinal wall damage.^[^23^,^^24^^]^ In this study, sepsis is a risk factor for NEC in infants with LBW, which is consistent with the results of previous related studies. However, it is been reported that sepsis due to other source of infection such as birth injury is a risk factor for NEC.^[25]^ Newborns grow fast, and the expression of intestinal vascular growth factor is enhanced by the stimulation of sepsis.^[26]^ When concentrated red blood cells are infused, it can cause blood redistribution in the body and change the blood supply of the mesentery.^[27]^ The infused histocompatibility antigen can cause immune response and damage. In the intestinal mucosal barrier, large amounts of oxygen free radicals can also cause reperfusion injury.^[^28^,^^29^^]^ This study has found that breastfeeding is a protective factor for NEC, which is consistent with the results of previous related studies.^[^23^,^^30^^]^ Breast milk contains high secretory IgA, lactoferrin, peroxidase and anti-inflammatory factors, which can enhance the immunity of newborns and promote the proliferation of intestinal bifidobacteria.^[^31^,^^32^^]^ Probiotics can regulate the balance of intestinal flora, neutralize intestinal toxins, and promote the decline of harmful bacterial enzyme activities, activate the immune function of macrophages, neutrophils and natural killer cells, and promote the repair and enhancement of intestinal mucosal epithelial cells function, thereby reducing the occurrence of neonatal NEC.^[^33^–^35^]^

Sepsis can cause large-scale growth and reproduction of many kinds of bacteria in the blood, and produce a large amount of toxins that enter the intestine through the blood circulation and destroy the body's negative feedback function, leading to intestinal mucosal necrosis.^[^36^,^^37^^]^ The results of multiple studies have shown that sepsis is a risk factor for neonatal NEC. Breastfeeding is a protective factor in the occurrence of NEC in LBW infants. The nutritional value of breast milk is more in line with the needs of newborns.^[33]^ The protective ingredients in breast milk can effectively improve the intestinal flora, promote the growth of beneficial bacteria, and significantly reduce the occurrence of NEC.^[^38^,^^39^^]^ It is been reported that infants are prone to NEC within 24 hours of blood transfusion, because blood transfusion therapy can change the blood distribution, resulting in a significant increase in intestinal free radicals and causing reperfusion injury.^[40]^ The results of this study indicate that interventions should be made as soon as possible for NEC in LBW infants to improve the prognosis.

Anti-inflammatory, antispasmodic and other medical treatments are difficult to achieve satisfactory results for children with NEC who have full-thickness mucosal pathological changes and hardening of the intestinal wall.^[41]^ At this time, children with intestinal inflammation may further involve normal adjacent tissues, and surgical treatment should be performed in time.^[42]^ The principle of surgical treatment is to carry out timely surgical intervention as far as possible before the intestinal ischemic necrosis of the infants has not been perforated. The timing of surgery associated with NEC is difficult for clinicians to grasp in clinical NEC diagnosis and treatment. At present, it has been believed that the absolute indication for surgery is a plain radiograph of the abdomen that prompts "pneumoperitoneum, X-rays suggest that portal vein gas and thrombocytopenia, severe acidosis and shock and other progressive clinical symptoms and signs are also relative surgical indications.^[^43^–^45^]^ For infants with free air under the diaphragm found in plain radiographs of the abdomen, unstable hemodynamics and aggravated abdominal distension, surgical treatment should be performed in a timely manner to improve the prognosis of the infants.^[46]^

## 5 Conclusions

To sum up, we have found that the NEC in LBW infants is very common, and congenital heart disease, sepsis, and blood transfusion were the independent risk factors for NEC in LBW infants, and breastfeeding, probiotics feeding were the protective factors for the NEC in LBW infants. It is necessary to actively intervene against congenital heart disease, sepsis and blood transfusions as risk factors for NEC. Besides, breastfeeding should be highly advocated, and probiotics are given to children to adjust the balance of intestinal flora.^[47]^ Limited by small sample size, future studies with larger sample size and rigorous design are needed to further elucidate the potential risk factors of NEC in infants, to provide reliable evidences to the clinical prevention and treatment of NEC.

## Author contributions

**Conceptualization:** Xuerong Tan.

**Data curation:** Xuerong Tan.

**Formal analysis:** Xuerong Tan.

**Investigation:** Xuerong Tan, Yunxia Zhou, Li Zhang, Jiaying Wang.

**Methodology:** Xuerong Tan, Jiaying Wang, Wenqiong Yang.

**Project administration:** Lan Xu, Wenqiong Yang.

**Resources:** Xuerong Tan, Lan Xu, Wenqiong Yang.

**Software:** Yunxia Zhou, Lan Xu.

**Supervision:** Yunxia Zhou, Jiaying Wang.

**Validation:** Xuerong Tan, Yunxia Zhou, Li Zhang.

**Visualization:** Xuerong Tan, Li Zhang.

**Writing – original draft:** Xuerong Tan, Li Zhang.

**Writing – review & editing:** Xuerong Tan.
